# The structure function as new integral measure of spatial and temporal properties of multichannel EEG

**DOI:** 10.1007/s40708-016-0040-8

**Published:** 2016-02-25

**Authors:** Mikhail Trifonov

**Affiliations:** IM Sechenov Institute of Evolutionary Physiology and Biochemistry of the Russian Academy of Sciences, Saint-Petersburg, Russia

**Keywords:** Multichannel EEG, Structure function, Variogram, Scaling law, EEG absolute increment distribution

## Abstract

The first-order temporal structure functions (SFs), i.e., the first-order statistical moment of absolute increments of scaled multichannel resting state EEG signals in healthy children and teenagers over a wide range of temporal separation (time lags) are computed. Our research shows that the sill level (asymptote) of the SF is mainly defined by a determinant of EEG correlation matrix reflecting the EEG spatial structure. The temporal structure of EEG is found to be characterized by power-law scaling or statistical-scale invariance over time scales less than 0.028 s and at least by two dominant frequencies differing by less than 0.3 Hz. These frequencies define the oscillation behavior of the SF and are mainly distributed within the range of 7.5–12.0 Hz. In this paper, we propose the combined Bessel and exponential model that fits well the empirical SF. It provides a good fit with the mean relative error fitting of 2.8 % over the time lag range of 1 s, using a sampling interval of 4 ms, for all cases under analysis. We also show that the hyper gamma distribution (HGD) fits to the empirical probability density functions (PDFs) of absolute increments of scaled multichannel resting state EEG signals at any given time lag. It means that only two parameters (sample mean of absolute increments and relevant coefficient of variation) may approximately define the empirical PDFs for a given number of channels. A three-dimensional feature vector constructed from the shape and scale parameters of the HGD and the sill level may be used to estimate the closeness of the real EEG to the “random” EEG characterized by the absence of temporal and spatial correlation.

## Introduction

Electroencephalogram (EEG) signals or EEG time series are the ones of the basic methods for analysis of brain activity in health and disease [1]. However, it is not yet fully known now how the EEG activity recorded at any location on the human scalp is formed. With a certain EEG signal, one can only guess something about the behavior of the underlying neuronal elements, but nobody can precisely reconstruct it since the relevant inverse problem does not have a unique solution. The absence of adequate physiological or mathematical ideas of EEG generation stimulates researchers to analyze EEG time series using various algorithms based on the concepts of different theories where the progress is evident. Since the foundations of these theories are fundamentally different, one can get a variety of descriptive measures concerning the same EEG signal. The most popular measures used in EEG time series analysis [2] came from the information theory [3], non-linear dynamics, and deterministic chaos theory [4].

Fulcher et al. [5] found that there are now over 9000 methods for time series analysis, which quantify a wide range of time series properties. Actually, a reduced set of 200 methods, including autocorrelation, auto-mutual information, stationarity, entropy, long-range scaling, correlation dimension, wavelet transforms, linear and non-linear model fits, and measures from the power spectrum, is enough to form a concise summary of the different behaviors of time series analysis methods applied to scientific time series [5].

It should be noted that the various entropy methods that are very popular in EEG time series analysis [1, 6, 7] like Sample Entropy (SampEn) [8], Lempel–Ziv complexity [9], auto-mutual information, Shannon’s entropy, and other approximate entropies are most similar according to Fulcher et al. [5] to the approximate entropy algorithm (ApEn) proposed by Pincus [10]. Unfortunately, current entropy measures are mostly unable to quantify the complexity of any underlying structure in the series, as well as determine if the variation arises from a random process [11]. Since a high entropy score indicates a random or chaotic series, and a low score indicates a high degree of regularity [11], it would be better to use them, at least just, for now, only for comparisons between different conditions (e.g., resting vs. task) or systems (e.g., young vs. old) as was suggested in [12].

The presence of chaos in time series is investigated through the correlation dimension *D*_2_ [13] and Lyapunov exponent (LE) [14] methods. The *D*_2_ reflects the self-similarity, and the maximal LE reflects the predictability. Zang et al. [15] found that both are associated with harmonic content in the time series. The fractional or scaling property is studied in terms of the Hurst exponent [16] and Rényi dimension [17].

Recent detailed examination of commonly used complexity measures from information theory, chaos theory, and random fractal theory for characterizing EEG time series shows that their variations with time are either similar or reciprocal, but the behaviors of some of them are counter-intuitive and puzzling [2]. It is not surprising, and one has to be careful with using various complexity measures adopted from theories mentioned above for EEG analysis. There are at least two reasons for that.

The first one is that the brain is not completely deterministic, and the stochasticity may influence its function in some cases [18]. Furthermore, time series arising from chaotic systems share with those generated by stochastic processes several properties like a wide-band power spectrum, a delta-like autocorrelation function, and an irregular behavior of the measured signals that make them very similar and, as a result, the distinction between them is not trivial [19]. Indeed, the *D*_2_ values and ‘maximum likelihood estimate of the correlation dimension’ (DML value) found in [20] for the white (gamma, uniform, Gaussian, and k-distributed) and correlated stochastic time series consisted of 50,000 data points which suggest that these data have a low fractal dimension which might be interpreted as the presence of chaos. The LE estimated for the same time series also suggests this interpretation [20]. It would be better to use the *D*_2_, the DML, and the LE, at least just, for now, for comparisons between different conditions (e.g., resting vs. task) or systems (e.g., young vs. old) as was suggested in [12] for using entropy measures.

The second reason is that a basic requirement for using measures adopted from non-linear dynamics and chaos theory implies the stationarity in the EEG time series, but it is not the case. Even detrended fluctuation analysis (DFA) specifically introduced by Peng et al. [21] to address non-stationarity estimates can be affected by very simple sinusoidal periodicities [22] or large-amplitude transient artifacts [23].

To avoid any speculations about what types of deterministic and/or stochastic processes govern the EEG signals, we will use here some properties of these signals that depend on processes underlying them but do not require knowing their exact nature. The one-dimensional probability density functions (PDFs) of the absolute increments of scaled multichannel resting state EEG signals calculated over a wide range of temporal separation (time lags) may be used as one of the properties [24]. It is not sufficient to infer the EEG dynamics, but it is enough to capture some of its features. It should be noted that the moment of order *p* of the relevant distribution represents the structure functions (SFs) of order *p* at a given time lag. The term “structure function” as such was proposed by Obukhov [25] but Kolmogorov [26, 27] was the first to introduce the formal definition of the second- and third-order SFs under a theoretical analysis of velocity difference of a turbulent fluid. Kolmogorov’s second-order SF is also known today as the variogram. The latter has been widely used for many years to quantify the spatial variability of spatial phenomena for many years in geostatistical studies (e.g., Gringarten and Deutsch [28]) as well as to describe a pseudo-periodic signal [29]. It is important that the SF approach is applicable to non-stationary time series. It does not require the mean estimation and is one of the several techniques available for calculating the generalized Hurst exponent.

Since the EEG absolute increment distributions are non-Gaussian [24], it is reasonable to use the SFs of various orders to analyze EEG data. It is more applicable than restricting the analysis using the autocorrelation function only. There are just a few examples of using variograms and SFs in neurosciences. The first attempt to investigate the brain’s electrical activity by deriving the second-order temporal SF for every separate electrode that appears was performed by Sergeev et al. in 1968 [30]. The application of the variogram technique for analysis of fundamental brain waves, as recorded by the EEG, was done by Conte et al. [31]. In their research, records from only four electrodes (CZ, FZ, OZ, and T4) were used and the multivariate variogram was calculated for the time series formed as the Euclidean norm of these four records. Timashev et al. [32] used the scale of fluctuations in the difference moment of the second-order SFs as an objective diagnosis of psychiatric disorders, such as schizophrenia, by analyzing the EEG signals recorded from scalp-mounted frontal electrodes at locations F3 and F4. The example of the first-order temporal SF calculated for the scaled EEG time series formed as the Euclidean norm of sixteen EEG signals was presented in [24]. Recently, Sleimen-Malkoun et al. [12] used a battery of multiscale metrics, including variogram to investigate the changes of cortical dynamics with aging. Chernyavskiy et al. [33] reported a successful attempt to classify EEG data of subjects with traumatic brain injury symptoms on the base of the spatiotemporal variogram computed from their EEG. In the latter case, the variogram is formally considered as isotropic but the authors said nothing about the number of electrodes (spatial sampling points) they used. This is important since for reliable estimation of two-dimensional isotropic variogram, Webster and Oliver [34] recommend ideally having 150 sampling points. For comparison, a minimum of some 30–50 increments is needed to estimate the one-dimensional variogram reliably as suggested in [35]. In this case, the variogram is only calculated for values of 15–25.

All results of the application of variograms and SFs for analysis of EEG time series mentioned above are very promising, and the studies in this area need to be continued. The present paper provides the detailed analysis of multivariate temporal SF of multichannel EEG. Generally, the SF may exhibit complex behavior over time lag and very short EEG time series may be not enough for its analysis. It imposes the limit on the applicability of the method.

This paper is organized as follows: the EEG data and the proposed method of their analysis are introduced in Sect. 2. Section 3 presents the results of the EEG analysis. The conclusions are drawn in Sect. 4. In the same section, the suggestions for further studies are proposed.

## The methods

### EEG data collection

**Fig. 1:**
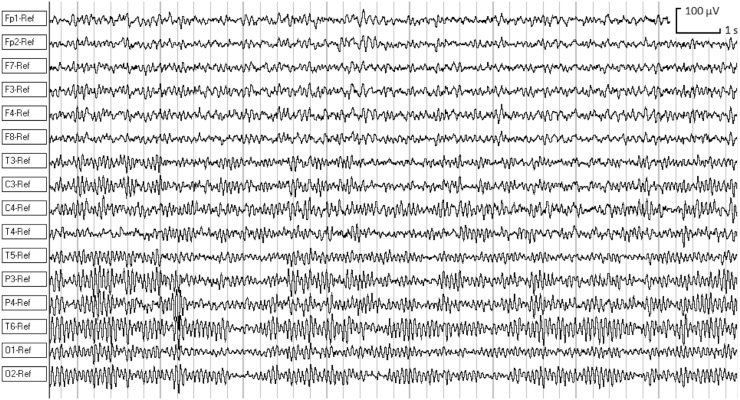
An example of real 16-channel EEG signals for subject *s*1. The channels are labeled as Fp1, Fp2, F7, F3, F4, F8, T3, C3, C4, T4, T5, P3, P4, T6, O1, and O2. Figure in the *top right corner* shows the 100 μV amplitude (*vertical line*) and 1 s duration (*horizontal line*) of the EEG record

90 cases of the eye closed resting state EEG signals were obtained for several years from healthy children and teenagers aged 5–19.8 using computer-aided electroencephalography analyzer Entsefalan-131-03 (Medikom, Russia) in 16 channels with sampling frequency *F*_d_ of 250 Hz. 16 Ag/AgCl electrodes were placed at Fp1, Fp2, F3, F4, F7, F8, C3, C4, P3, P4, T3, T4, T5, T6, O1, and O2 on the scalp according to the international 10–20 system. The option of linked earlobes was used as a reference for a montage. The written permission from parents for all subjects to participate in the study was obtained. Only artifact-free epochs with a length longer than 36 s have been selected for further analysis. An example of real 16-channel EEG fragment recorded from a subject *s*1 is shown in Fig. 1 where 100 μV amplitude scale (vertical line) and 1 s duration (horizontal line) are shown in the top right corner.

### EEG data analysis

In this research, we used the method of the EEG signals analysis briefly presented in our previous paper [4]. Some relevant parts of it are reproduced below to help the understanding of the results given in Sect. 3.

Let {***X***(*t*) = [*X*_1_(*t*), *X*_2_(*t*), …, *X*_*m*_(*t*)]^T^, *t* = 1, 2,…, *N*} represent the observed EEG *m*-dimensional time series, where *m* is a number of channels (electrodes), here equal to 16, ***X***(*t*) is an *m*-dimensional vector*, X*_*j*_(*t*) is the signal amplitude on the channel *j* expressed in microvolts at the discrete integer valued time (sampling point) *t*, *N* is the length of series, and the superscript T denotes the matrix transpose operation. The sampling interval (in seconds) equates to 1/*F*_d_.

Since signals reveal significant spread of amplitude values from subject to subject the original EEG time series is centered by subtracting their mean in every channel first and then scaled by the [det(***Σ***_***X***_)]^(1/2m)^, where det denotes the determinant, ***Σ***_***X***_ = *E*[*δ* ***X*** *δ* ***X*** ^T^] is the sampling covariance matrix, *δ* ***X*** = ***X*** − *E*[***X***], and *E*[·] denotes the statistical expectation or average. As a result, any new vector ***Y***(*t*) = *δ* ***X***(*t*)**/**[det(***Σ***_***X***_)]^(1/2m)^ is dimensionless and has the same generalized variance independently on the subject since the determinant of the covariance matrix ***Σ***_***Y***_ = *E*[*δ* ***Y*** *δ* ***Y*** ^T^] is equal to 1. Geometrically, the quantity [det(***Σ***_***Y***_)]^1/2^ determines the volume of the confidence ellipsoid for any particular confidence level, and the scaling proposed here makes the distributions of any vector ***Y*** to be equivalent in the sense that they occupy the same volume in *m*-dimensional space. It means that the ellipsoids with different orientations and different semi-axes but having the same generalized variance will be considered equivalent.

Define the absolute increment (relative displacement) Δ*Y*_*τ*_ as the Euclidean distance between two vectors ***Y***(*t*) and ***Y***(*t* + *τ*) which are separated by a dimensionless and relative lag parameter *τ* = 1, 2…, *N* − *τ*

1 $$\Delta Y_{\tau } = \left| {\varvec{Y}\left( {t + \tau } \right) - \varvec{Y}(t)} \right| = \sqrt {\mathop \sum \limits_{j = 1}^{m} \left( {Y_{j} \left( {t + \tau } \right) - Y_{j} (t)} \right)^{2} } .$$

The actual time difference (in seconds) between samples ***Y***(*t* + *τ*) and ***Y***(*t*) is calculated as the time lag *τ* divided by the sampling frequency *F*_d_, i.e., equal to *τ*/*F*_d_.

Recently, Trifonov and Rozhkov [24] have analyzed the PDFs of Δ*Y*_*τ*_ and found them closely matched to hyper gamma distribution (HGD) proposed by Suzuki [36]

2 $$f\left( {\Delta Y_{\tau } } \right) = \gamma \Delta Y_{\tau }^{v - 1} \exp \left( { - \beta \Delta Y_{\tau }^{\alpha } } \right),$$

where *γ* = *αβ* ^*ν*/*α*^/Γ(*ν*/*α*), Γ(·) is the gamma function, *α* and *ν* are shape parameters, and *β* is the scale parameter. Generally, all these parameters may depend on *τ*, but for simplicity of notation the index *τ* is omitted from them.

The choice of given type of fitting function was based on the fact that the “random” EEG absolute increment Δ*Y*_*τ*_ (obtained from *m*-dimensional vector ***X***(*t*) whose components are independent normal random variables having zero mean and variance *σ* ^2^) is distributed according to the scaled chi-distribution that is a special case of (2) when *ν* = *m*, *α* = 2, and *β* = 0.25/*σ* ^2^. It should be noted that both the HGD and the scaled chi-distribution are special cases of the Amoroso distribution [37]. The HGD is closed under scaling by a positive factor, and the random variable (*E*[Δ*Y*_1_]/*E*[Δ*Y*_*τ*_])·Δ*Y*_*τ*_ has the same distribution as Δ*Y*_1_.

Letting *ν* be *m*, and solving a single non-linear equation

$$\left( {{{\sigma_{\tau } } \mathord{\left/ {\vphantom {{\sigma_{\tau } } {E[\Delta Y_{\tau } ]}}} \right. \kern-0pt} {E[\Delta Y_{\tau } ]}}} \right)^{ 2} = \varGamma \left( {{{\left( {m + 2} \right)} \mathord{\left/ {\vphantom {{\left( {m + 2} \right)} \alpha }} \right. \kern-0pt} \alpha }} \right) \, \varGamma \left( {m/\alpha } \right)/ \, \left[ {\varGamma \left( {{{\left( {m + 1} \right)} \mathord{\left/ {\vphantom {{\left( {m + 1} \right)} \alpha }} \right. \kern-0pt} \alpha }} \right)} \right]^{ 2} {-}{ 1},$$

where *σ*_*τ*_ is the sample standard deviation one can estimate the value of parameter *α*. The scale parameter *β* is estimated then as

$$\beta = {{\left[ {\varGamma \left( {{{\left( {m + 1} \right)} \mathord{\left/ {\vphantom {{\left( {m + 1} \right)} \alpha }} \right. \kern-0pt} \alpha }} \right)} \right]^{\alpha } } \mathord{\left/ {\vphantom {{\left[ {\varGamma \left( {{{\left( {m + 1} \right)} \mathord{\left/ {\vphantom {{\left( {m + 1} \right)} \alpha }} \right. \kern-0pt} \alpha }} \right)} \right]^{\alpha } } {\left[ E \right[\Delta Y_{\tau } ]\varGamma \left( {m/\alpha } \right)]^{\alpha } }}} \right. \kern-0pt} {\left[ E \right[\Delta Y_{\tau } ]\varGamma \left( {m/\alpha } \right)]^{\alpha } }}.$$

Since the EEG absolute increment distributions are non-Gaussian, a basic tool for Δ*Y*_*τ*_ analysis may be given by the *p*th-order SF *S*_*p*_(*τ*) which is defined as the expectation of the *p*th moment of Δ*Y*_*τ*_

3 $$S_{p} \left( \tau \right) = \frac{1}{N - \tau }\mathop \sum \limits_{t = 1}^{N - \tau } \Delta Y_{\tau }^{p} , \quad \tau = 1, \ldots .\tau_{max} ,$$

where *τ*_max_ is maximal time lag value. At least for *p* = 2 (the SF *S*_2_(*τ*) represents well-known multivariate variogram originally developed for spatial data analysis by Bourgault and Marcotte [38]) there is an upper limit for *τ*_max_. According to Petersen and Esbensen [39], *τ*_max_ should not be higher than *N*/2 (rounded down).

In practical, data analysis order value *p* may range from 1 to at most 10 or so [40]. However, according to Fisher et al. [41], the first-order SF is more robust than higher-order SFs with respect to outliers in the absolute increment. This conclusion may be partly illustrated by comparing the coefficients of variation (CV) of the first- and the second-order SF in the limiting case of “random EEG.” Since the random variable Δ*Y*_*τ*_ is distributed according to the scaled chi-distribution, while Δ*Y*_*τ*_^2^ is distributed according to the scaled *χ* ^2^ distribution, then one can easily derive analytical expressions for CV_1_ and CV_2_ as follows:

$${\text{CV}}_{ 1} = {\text{sqrt}}\left[ {{{0. 5m\cdot\varGamma^{ 2} \left( {m/ 2} \right)} \mathord{\left/ {\vphantom {{0. 5m\cdot\varGamma^{ 2} \left( {m/ 2} \right)} {\varGamma^{ 2} \left(\left( {m + 1} \right)/ 2\right)}}} \right. \kern-0pt} {\varGamma^{ 2} \left(\left( {m + 1} \right)/ 2\right)}}} - 1 \right],\quad {\text{ and}}\;{\text{CV}}_{ 2} = {\text{sqrt}}\left( { 2/m} \right).$$

Using these expressions, one can get that the ratio CV_1_/CV_2_ is slightly above 0.5 even for small *m* value. Preliminary analysis of real EEG data shows that using *S*_1_ is preferable as having the smallest coefficient of variation CV_1_ as well. For this reason, in this study, we concentrate our attention on and discuss the first-order SF only.

## The results

**Fig. 2:**
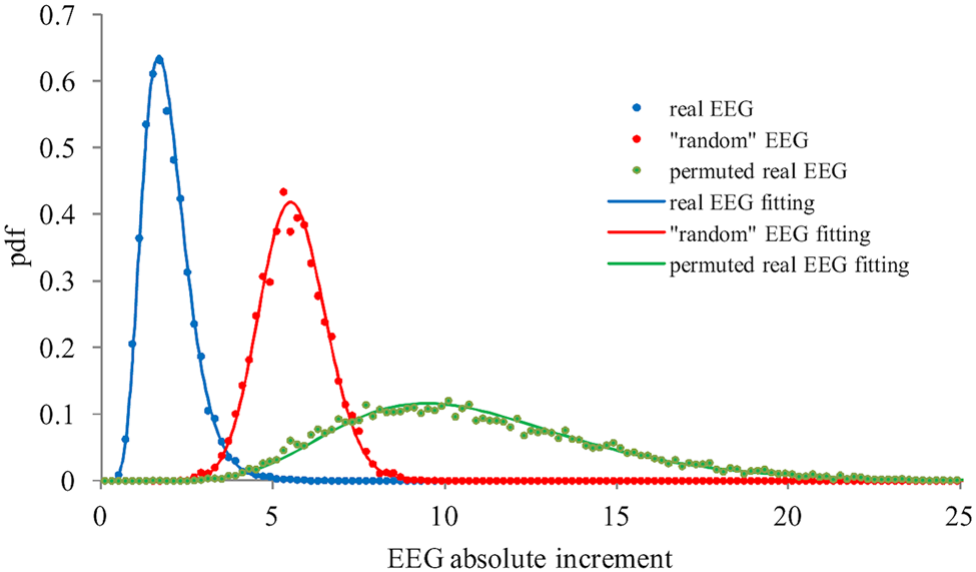
The examples of the empirical PDFs *f*_0_(Δ*Y*_1_) and HGD fits *f*(Δ*Y*_1_) for the cases of real (*blue* *α* = 0.51, *β* = 22.7), real, but randomly permuted (*green* *α* = 0.54, *β* = 8.3), and “random EEG” time series (*red* *α* = 2.01, *β* = 0.246), *ν* is everywhere 15

An example of the individual empirical PDF *f*(Δ*Y*_1_) and its HGD fit *f*(Δ*Ŷ*_1_) is shown in Fig. 2. In this case, the value of *N* is equal to 9000 and corresponds to 36-s EEG record with the sampling frequency *F*_d_ = 250 Hz. Time lag *τ* = 1 corresponds to the sampling interval of 0.004 s and the histogram bin size *W*_1_ = 0.2. For comparison, the PDFs for the “random” EEG with *σ*_*n*_ = 1 and for the original EEG with randomly permuted temporal sequence of time series ***X***(*t*) are shown in the same Fig. 2. Random permutation of real EEG time series and generation of the “random” EEG time series was done in Matlab using *randperm* and *randn* functions, respectively. Visually, all three theoretical HGD *f*(Δ*Ŷ*_1_) provide a reasonably good fit for all three empirical PDF *f*(Δ*Y*_1_).

The closeness of the empirical normalized histogram Δ*y*_1_ = *W*_1_·Δ*Y*_1_ (*Σ*_*i*_Δ*y*_1*i*_ = 1) to the fitting normalized histogram Δ*ŷ*_1_ was checked by using Bhattacharyya coefficient *BC*(Δ*y*_1_, Δ*ŷ*_1_) = *Σ*_*i*_sqrt(Δ*y*_1*i*_·Δ*ŷ*_1*i*_) as a measure of similarity between them [42]. It was found that this coefficient equates to 0.999 for the HGD fitting to the empirical distribution in the case of real and “random” EEG time series and 0.997 in the case of real, but randomly permuted EEG time series. For comparison, the closeness of real and “random” EEG normalized histograms is about 0.147.

The same approach was used to check the closeness between the empirical normalized histograms Δ*y*_1_ and Δ*ŷ*_1_ = (*E*[Δ*Y*_1_]/*E*[Δ*Y*_*τ*_])·*W*_*τ*_·Δ*Y*_*τ*_ at different time lags *τ* under scaling mentioned above. We found that for randomly selected real EEG time series, and for time lags *τ* up to 100, this coefficient oscillates between 0.994 and 1 about a mean value of 0.997. It means that empirical distribution of Δ*Y*_*τ*_ is really closed under the scaling and has approximately the same parameters *α* and *ν* as the distribution of Δ*Y*_1_ has. This result allows us to use empirical PDFs estimated at unity time lag only in our further analysis.

Estimating *α* and *β* parameters at *τ* = 1 according to the approach mentioned above, we have found that their values are in the range [0.30, 0.71] and [12.28, 45.67], respectively. The analysis of the behavior of these parameters allows us to make a suggestion that they are not completely independent and that α decreases approximately exponentially with *β*. We could not test this suggestion carefully because we have not found yet empirical PDFs with extremely high values of *β* which may imply that real EEG becomes strongly deterministic. It seems we need to estimate *α* and *β* in this case theoretically as it was done in another extreme case of “random” EEG (in the sense defined above) when we get *α* = 2 and *β* = 0.25. The latter two parameters may serve as a reference point and the distance to it from any point on the ***αβ*** diagram can be used as a quantitative measure of the degree of temporal randomness in real EEG.

The values of the *BC* values for the whole set of 90 real EEG time series range between 0.994 and 0.999 with the average value of 0.998 and standard deviation of less than 0.001. Such result indicates that the HGD provides a good fit to the empirical PDFs. The closeness of any real EEG normalized histograms from our dataset to the “random” EEG normalized histogram expressed in terms of the *BC* value is in the range [0.013, 0.621].

**Fig. 3:**
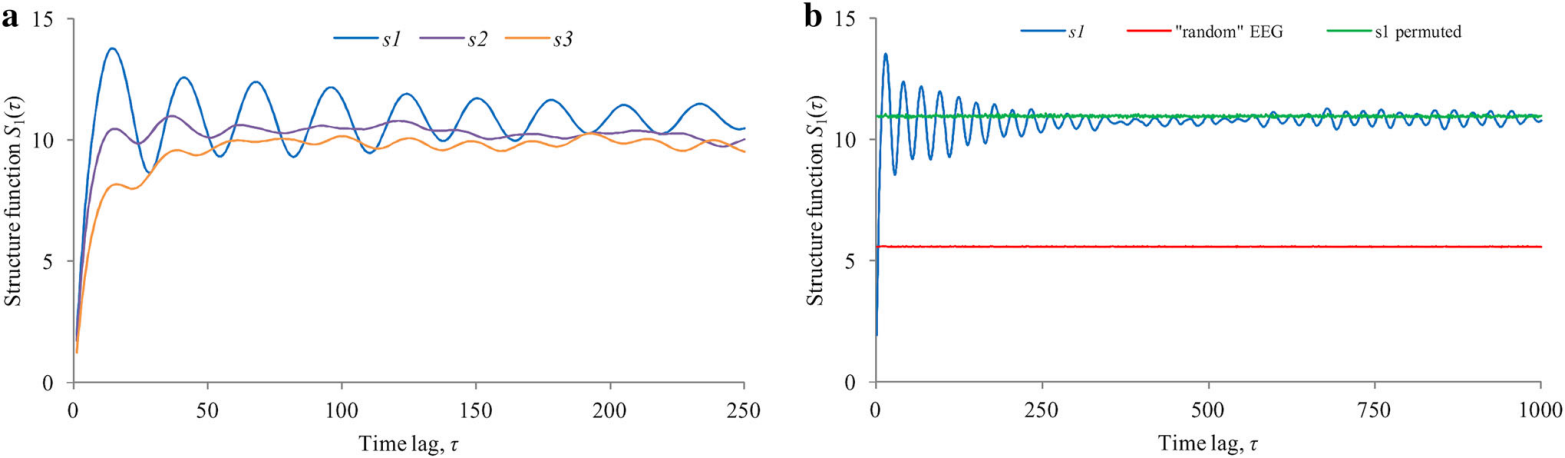
**a** The examples of the first-order SF *S*_1_(*τ*) derived from the three real EEG time series (subjects *s*1, *s*2, and *s*3). **b** *S*_1_(*τ*) corresponding to the subject *s*1 [real EEG—(*blue*), real but randomly permuted EEG—(*green*)], and *S*_1_(*τ*) derived from “random” 16-channel EEG time series (*red*)

The empirical first-order SFs over the *τ*_max_ = 1000 (*N* = 9000) were calculated using Eq. (3). The three typical examples of *S*_1_(*τ*) with lag *τ* up to 250 corresponding to the 16-channel real EEG time series, are shown in Fig. 3a. Figure 3b represents *S*_1_(*τ*) for subject *s*1 (in the case of real EEG and its randomly permuted version), and for “random” EEG. The randomly permuted EEG means disregarding the temporal order of the original EEG time series in all channels simultaneously keeping the mean vector *E*[***X***] and the sampling covariance matrix ***Σ***_***X***_ unchanged. It is obvious that such permutation keeps the original inter-channel (spatial) correlation structure of any EEG time series, but destroys original temporal correlation within each channel.

Actually, Fig. 3a shows that the individual first-order SF *S*_1_(*τ*) increases approximately exponentially with increasing time lag *τ* for the first few *τ* values, but starting with some τ* value the *S*_1_(*τ*) begins to oscillate around its sill level *V* rather than approaches it monotonically. In geostatistical studies, the variogram (the second-order SF) exhibiting cyclicity (under-dumped behavior) has been termed a “hole-effect variogram” (e.g., Ma and Jones [43]). Such behavior means that there is a high temporal correlation between a given EEG sample and neighboring EEG samples as well as some relatively distant EEG samples. The curve *s*1 in Fig. 3a exhibits strong cyclicity while curves *s*2 and *s*3 exhibit low cyclicity which corresponds to a higher damping ratio in terms of under-damped motion.

At the same time, both randomly permuted real EEG, and “random” EEG should not reveal significant temporal correlation by definition, and one can see that the relevant *S*_1_(*τ*) does not really depend on *τ*. They differ only in the sill level *V*. The first one is around the sill value corresponding the original EEG while the second one is close to the theoretical value *V*_0_ of 5.569 corresponding to the “random” EEG time series for *σ*_*n*_ = 1 and *m* = 16. These levels appear to be defined by the type of spatial correlation characterized by the sampling correlation matrix ***R***_***X***_. According to Peña and Rodríguez [44], the determinant of ***R***_***X***_ summarizes the linear relationships between the variables and may be used as the scalar measure of multivariate linear dependence. Proposed in [44] measure named as effective dependence is defined by

$$D_{\text{e}} \left( X \right) \, = { 1 } - \, \left[ {{ \det }\left( {R_{X} } \right)} \right]^{{ 1/{\text{m}}}} .$$

It was found here that there is an empirical dependence between *D*_*e*_ and the sill *V* which can be fitted by the non-linear expression

$$V \, = \, V_{0} /(1 \, - \, D_{e} )^{q} .$$

The value of *V*_0_/*V* may serve as a measure of randomness of the spatial structure of real EEG.

**Fig. 4:**
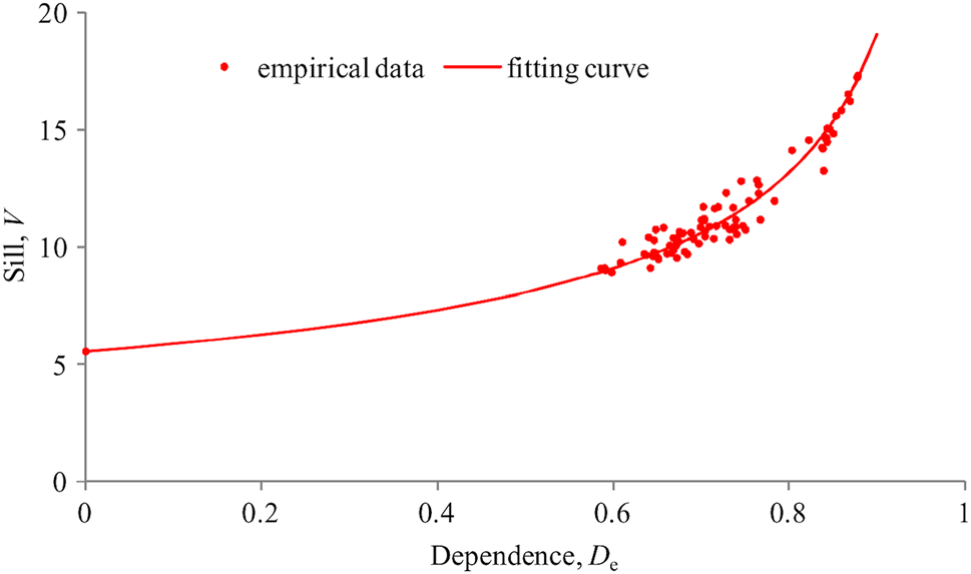
The empirical dependence between *D*_*e*_ and *V*

The empirical dependence between *D*_e_ and *V* derived from 90 cases of the eyes closed resting state EEG time series is shown in Fig. 4. The fitting parameter *q* is equal here to 0.535. (The coefficient of determination *R* ^2^ = 0.936 in the relevant log–log regression model). These findings approve the suggestion that the spatial EEG structure define the sill level of the first-order SFs. On the other hand, the temporal EEG structure is reflected in the under-damped oscillatory behavior of *S*_1_(*τ*). It was found that this behavior depends on the subject’s individuality and appears most conspicuous in the resting state. As usual, the dominant oscillation frequency lies in the alpha range. The amplitudes of peaks and troughs attenuate with increasing lag distance and the sill level *V* here is only meaningful at the larger time lags, where vectors ***X*** (and ***Y***) no longer exhibit any significant temporal correlation. The time lag *τ*_0_ at which the *S*_1_(*τ*_0_) reaches the sill value is usually called as range, but in practice, it is not rigorously defined. Theoretically, the expected range value *τ*_0_ should be equal to 0 for both first-order SFs corresponding to the randomly permuted real EEG and “random” EEG. And it is actually true as one can see from Fig. 3b.

It is reasonable to suggest that the cyclical pattern is typical for the spatial first-order SFs as well. This suggestion is not discussed because the number of channels is too small here, but it is indirectly supported by fMRI data analysis [45], where variograms waving behavior was found as the spatial lag distance increases.

It was found that all SFs *S*_1_(*τ*) derived from EEG time series appear to exhibit scaling, i.e., *S*_1_(*τ*) ∝ *τ* ^*ζ*^ at time lags *τ* ranging from 3 to 7 in dependence on the subject. To estimate the scaling exponent ζ, we used linear least squares fit to the SFs in log–log coordinates and *R*-squared method proposed by Pressel and Collins [46]. We let a lower bound on the coefficient of determination *R* ^2^ of 0.998 for our analysis. Such choice is explained here by a relatively low number of *S*_1_(*τ*) samples within the range between 1 and *τ*_0_, where *S*_1_(*τ*_0_) reaches (intersects) first the sill value *V*. Most likely by increasing the sampling frequency *F*_*d*_ one can identify more distinct scaling regions with the breaks in *S*_1_(*τ*) slope. It should be noted that according to [41, 46], the first-order SF scaling exponent ζ is simply the Hurst exponent *H* that has a clear physical meaning. We have found that the average value of ζ in our analysis is 0.88 ± 0.03 for time scales less than 0.028 s. It means that one-dimensional random process $|{X(t)}|$ (and $|{Y(t)}|$) is characterized at these scales by persistent increments and long-range correlations. Since the Hurst exponent of such a process is related to the fractal dimension *D* by *D* = 2 − *H*, then *D* = 1.12 and this process has a distinctly less space filling. Within the EEG dataset considered here, the scaling behavior slightly depends on subject and condition during the eyes closed resting state EEG is recorded.

We adopt the idea of using the Bessel model for describing the pronounced hole-effect structure in the variogram function proposed in [45]. We propose here to fit the combined Bessel and exponential model *S*_1_ *(*τ*) to the empirical first-order SF *S*_1_(*τ*). The model *S*_1_ *(*τ*) is defined as

4 $$S_{1}^{*} \left( \tau \right) = V\left\{ {1 - \left[ {\exp \left( { - \alpha_{0} \tau } \right) + 1 - \exp \left( { - \beta_{0} \tau } \right)} \right]\mathop \sum \limits_{k = 1}^{K} p_{k} J_{0} \left( {\omega_{k} \tau + \theta_{k} } \right)} \right\},$$

where *K* is the order of the model, *J*_0_(·) is the Bessel function of the first kind and zero order, *p*_*k*_ is weight (*p*_*k*_ = *w*_*k*_ */J*_0_(*θ*_*k*_), *w*_*k*_ ≥ 0*, Σ*_*k*_ *w*_*k*_ = 1), *ω*_k_ and *θ*_*k*_ are frequency and phase parameters, respectively, and *α*_0_ and *β*_0_ are positive constants (*α*_0_ > *β*_0_). It should be noted that the model (4) is valid only if the derivative [*S*_1_ *(*τ*)]′ ≤ 0 at *τ* → 0. This condition imposes a limitation on the choice of phase parameters *θ*_*k*_.

The beat pattern clearly pronounced in empirical *S*_1_(*τ*) for large time lags (see the curve *s*1 in Fig. 3b) can arise from interference between at least two cosine signals of slightly different frequencies. Since *J*_0_(*z*) for large argument *z* is just cosine (*J*_0_(*z*) ~ *cos*(*z* − π/4)), the model (4) should have at least order 2 for the case of strong cyclicity. The mean values of frequency and phase parameter *ω*_0_ and *θ*_0_ for such a model can be estimated from the matching of root locations for function *J*_0_(·) and *C*(*τ*) = (1 − *S*_1_(*τ*)/*V*) within the time lag *τ* range [0, 260]. If *R*_*J*0_ is a set of actual roots of *J*_0_(·) and *r*_S1_ is a set of roots of *C*(*τ*) estimated by linear interpolation, then ω_0_ and *θ*_0_ are evaluated as regression coefficients in the simple linear regression model

$$R_{J0}^{*} = \theta_{0} + \omega_{0} r_{S1} ,$$

where $R_{J0}^{*}$ is the estimate of the known *R*_*J*0_.

**Fig. 5:**
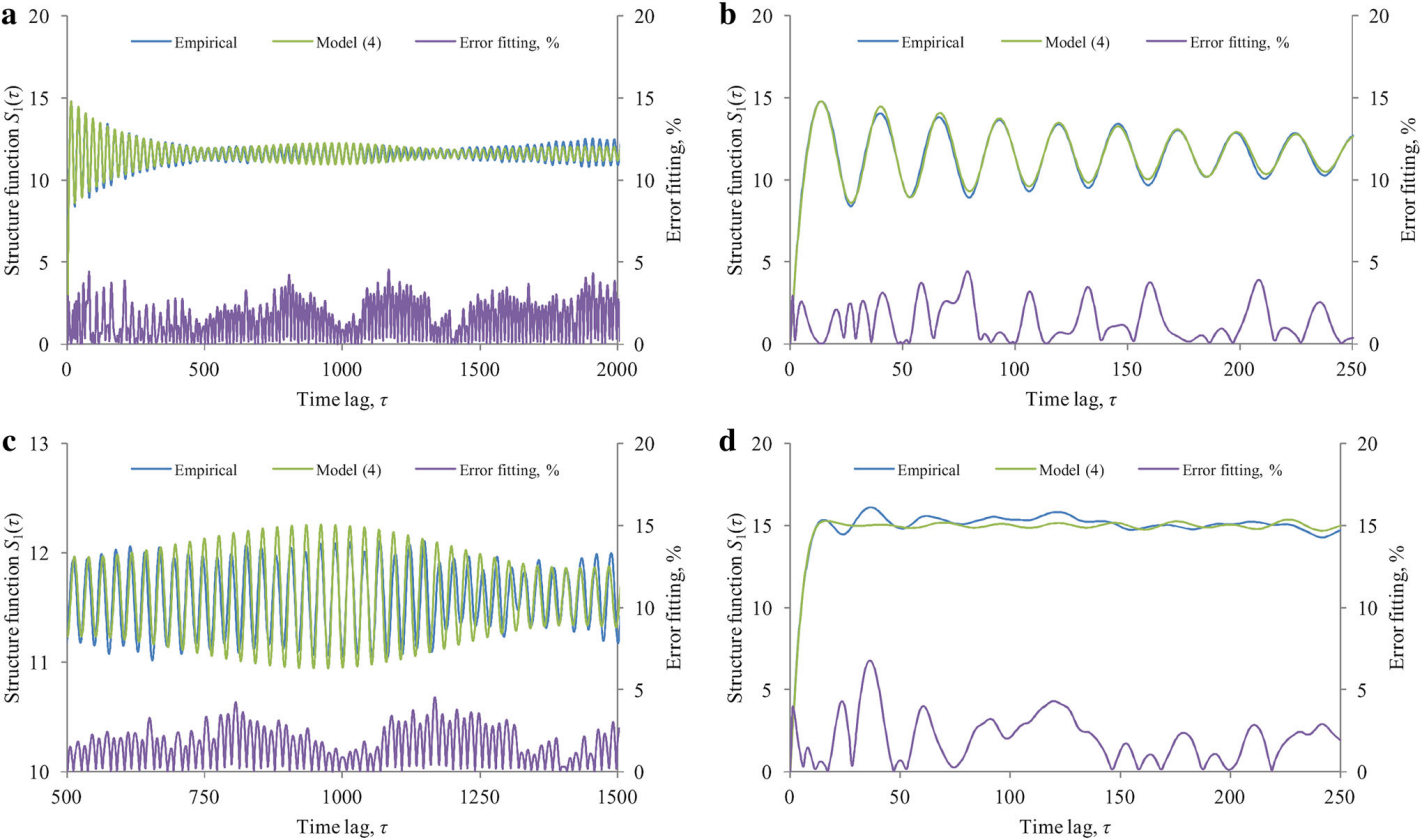
Two examples of the model (4) fitting to the empirical *S*_1_(*τ*): **a**–**c** Subject *sK*, 16 years old, (high cyclicity), *ω*_1_ = 0.239 (*f*_1_ = 9.51 Hz), *θ*_1_ = 0.6, *ω*_2_ = 0.232 (*f*_2_ = 9.23 Hz), *θ*_2_ = 0.95, *ω*_3_ = 0.245 (*f*_3_ = 9.75 Hz), *θ*_3_ = 0.05, *α*_0_ = 0.17, *β*_0_ = 0.054, *w*_1_ = 0.7, *w*_2_ = 0.2, *w*_3_ = 0.1; mean relative error fitting is 1.4 %; **d** Subject *sR*, 12 years old, (low cyclicity), *ω*_1_ = 0.239 (*f*_1_ = 9.51 Hz), *θ*_1_ = −0.05, *ω*_2_ = 0.157 (*f*_2_ = 6.25 Hz), *θ*_2_ = −0.05, *ω*_3_ = 0.129 (*f*_3_ = 5.13 Hz), *θ*_3_ = 0.15, *α*_0_ = 0.17, *β*_0_ = 0.001, *w*_1_ = 0.6, *w*_2_ = 0.2, *w*_3_ = 0.2, mean error fitting is 2 %; Error fitting = 100 % $|{S_{1}} (\tau)- {S_{1}^{*}}(\tau)|/{S_{1}} (\tau)$, mean relative error fitting is calculated over the time lag *τ* range of 1 s (*τ* $\in$ [0, 250]); [*S*_1_ *(0)]′ are everywhere ≥ 0

We tried to fit the model (4) with *K* = 3 to the empirical *S*_1_(*τ*). The examples of such a fitting are shown in Fig. 5. To find the estimates for model parameters, we used a simplified algorithm and estimate of *ω*_0_. At first, the frequencies *ω*_1_, *ω*_2_, and *ω*_3_ were estimated by finding the first three maximum values of the discrete form of integral $\int_{0}^{{\tau_{ \hbox{max} } }} {C(\tau )\cos \left( {\omega \tau } \right){\text{d}}\tau }$ calculated with frequency step Δ*ω* = 0.001. This approach is based on the Neumann’s addition theorem and the fact that $\int_{0}^{\infty } {J_{v} \left( {\omega \tau } \right)\cos \left( {\omega \tau } \right){\text{d}}\tau }$ = ∞ for any order *v*. The phases *θ*_1_, *θ*_2_, and *θ*_3_ were estimated by finding the maximum ∫*C*(*τ*)*J*_0_(*ω*_*k*_ *τ* + *θ*_*k*_)d*τ* by varying *θ*_*k*_ separately for *k* = 1, 2, and 3. After that the weights *w*_1_, and *w*_2_ from the range [0, 1] were chosen (*w*_3_ = 1 − *w*_1_ − *w*_2_) by exhaustive search with step Δ*w*_1,2_ = 0.1 using the Nash and Sutcliffe criterion or *F* value [47, 48]

5 $$F = 1 - \frac{{\mathop \sum \nolimits_{\tau = 1}^{{\tau_{ \hbox{max} } }} (S_{1} \left( \tau \right) - S_{1}^{*} \left( \tau \right))^{2} }}{{\mathop \sum \nolimits_{\tau = 1}^{{\tau_{ \hbox{max} } }} (S_{1} \left( \tau \right) - E\left[ {S_{1} } \right])^{2} }},$$

as the criterion of the goodness of fit. The same searching approach was used at the final step for finding parameters of *α*_0_ and *β*_0_ with step Δ*α*_0_ = 0.01 and Δ*β*_0_ = 0.001, respectively, under condition *α*_0_ > *β*_0_.

The approach mentioned above does not yield the optimal set of estimated parameters to the model (4). However, the mean error fitting defined as 100 %·*E* $\left[\left|{S_{1}} (\tau)- {S_{1}^{*}}(\tau)\right|/{S_{1}} (\tau) \right]$ is 2.8 % for over the time lag *τ* within the range [0, 250] for all cases under analysis. It was found that frequencies *f*_1_, *f*_2_, and *f*_3_ are mainly distributed within the range of 7.5–12.0 Hz (84.4 % of all cases) with two clearly pronounced modes located at 9.75 and 10.75 Hz. There is also a less pronounced mode located at 6.25 Hz corresponding to the frequency distribution within the range of 4.5–7.5 Hz (15.6 % of all cases). These frequency estimates may be considered as trivial but for the high correlation of 0.94 between two nearest frequencies and the difference about 0.3 Hz between them that is observed for roughly 70 % of all cases.

## Conclusions and future work

The analysis of the first-order SFs derived from 90 eye closed resting state EEG records in healthy children and teenagers shows that this function contains integral information about spatial and temporal EEG organization. It was found that the sill level (asymptote) *V* of the *S*_1_(*τ*) indicates how strong the correlations between channels take place and, therefore, reflects the EEG spatial organization. The shape *α* and scale *β* parameters of the HGD, successfully representing the empirical distribution of the EEG absolute increment, characterize the degree of temporal randomness in real EEG. It is reasonable to construct a three-dimensional feature vector FV = (*α*, *β, V*)^T^ which may be used to estimate the closeness of the real EEG to the “random” EEG (in the sense defined above). In the case of 16-channel EEG, the reference FV_R_ has components 2, 0.25 and 5.569, respectively.

The EEG temporal organization is also characterized by the scaling exponent ζ (or Hurst exponent *H* in our case) and at least by two frequencies defining the type of *S*_1_(*τ*) cyclicity around the sill level. The time scale where EEG records appear to exhibit scaling depends on subject varying between 0.012 s and 0.028 s. The average value of ζ (or *H*) in our analysis is 0.88 ± 0.03. It means that one-dimensional random process $|{X(t)}|$ at low time scales is characterized by persistent increments and long-range correlations. The mere fact that ζ has a value close to unity and relatively small coefficient of variation can be considered only preliminary. It calls for further investigations.

The next characteristic defining the temporal structure of EEG is the cyclicity of *S*_1_(*τ*). In 84.4 % of cases, the derived *S*_1_(*τ*) exhibited relatively high oscillation (under-damped behavior) around the sill level with frequencies mainly distributed within the range of 7.5–12.0 Hz. At least two dominant frequencies differing by less than 0.3 Hz were found within this range. This fact is rather interesting and needs to be understood.

It is shown that the combined Bessel and exponential low-order model can capture the behavior of the first-order SFs exhibiting high cyclicity. The third-order model provides the mean relative error fitting of 2.8 % over the time lag range of 1 s, using a sampling interval of 4 ms, for all cases under analysis.

Our future studies will focus on the analysis of EEG recorded at a higher sampling frequency to identify more distinct scaling regions with the breaks in the slope of the first-order SF. Using such data can allow us to understand more carefully underlying stochastic processes and to suggest appropriate improvements in the model (4). Assuming that *S*_1_(*τ*) might be a solution of some forced second-order ordinary differential equation it would be interesting to think about the possible form of such equation.
